# Demographic History, Genetic Load, and the Efficacy of Selection in the Globally Invasive Mosquito *Aedes aegypti*

**DOI:** 10.1093/gbe/evaf066

**Published:** 2025-04-04

**Authors:** Tyler V Kent, Daniel R Schrider, Daniel R Matute

**Affiliations:** Department of Ecology and Evolution, University of Chicago, Chicago, IL, USA; Department of Biology, College of Arts and Sciences, University of North Carolina, Chapel Hill, NC, USA; Department of Genetics, School of Medicine, University of North Carolina, Chapel Hill, NC, USA; Department of Genetics, School of Medicine, University of North Carolina, Chapel Hill, NC, USA; Department of Biology, College of Arts and Sciences, University of North Carolina, Chapel Hill, NC, USA

**Keywords:** genetic load, demography, *Aedes*, population structure, invasive species

## Abstract

*Aedes aegypti* is the main vector species of yellow fever, dengue, Zika, and chikungunya. The species is originally from Africa but has experienced a spectacular expansion in its geographic range to a large swath of the world, and the demographic effects of which have remained largely understudied. In this report, we examine whole-genome sequences from six countries in Africa, North America, and South America to investigate the demographic history of the spread of *A. aegypti* into the Americas and its impact on genomic diversity and deleterious genetic load. In the Americas, we observe patterns of strong population structure consistent with relatively low (but probably nonzero) levels of gene flow but occasional long-range dispersal and/or recolonization events. We also find evidence that the colonization of the Americas has resulted in introduction bottlenecks. However, while each sampling location shows evidence of a past population contraction and subsequent recovery, our results suggest that the bottlenecks in America have led to a reduction in genetic diversity of only ∼35% relative to African populations, and the American samples have retained high levels of genetic diversity (expected heterozygosity of ∼0.02 at synonymous sites). We additionally find that American populations of *aegypti* have experienced only a minor reduction in the efficacy of selection, with evidence for both an accumulation of deleterious alleles and some purging of strongly deleterious alleles. These results exemplify how an invasive species can expand its range with remarkable genetic resilience in the face of strong eradication pressure.

**Significance**

*Aedes* mosquitoes are important vectors of numerous human disease–causing pathogens (including Dengue, yellow fever, West Nile, Zika, and eastern equine encephalitis). *Aedes aegypti* is a successful invasive and has undergone rapid range expansions following anthropogenic activity. In this paper, we use whole-genome sequencing to understand the demographic events that follow the expansion of *A. aegypti* into the Americas. We find that even though the invading populations have experienced population bottlenecks associated with the invasion of the Americas, the impact of these events on genetic diversity and the efficacy of selection appear to be relatively modest. Thus, the American populations of this important vector species are poised to continue to rapidly adapt to ongoing and future control efforts.

## Introduction

Invasive species pose a threat to native species and ecosystems as well as human health and agriculture (Chornesky and Randall 2003; Paini et al. 2016; Dueñas et al. 2021). While many invasive species spread by displacing or outcompeting native species, many instead take advantage of underutilized niches, often similar to those in their home environments (Elton 1958; Baker and Stebbins 1965; Baker 1974; Sakai et al. 2001; Barrett 2015; Liu et al. 2020). One such pathway is developing an association with humans, either by exploiting anthropogenic changes to the ecosystem or directly through the evolution of human preference (Hulme-Beaman et al. 2016). By adapting to these novel environments, invasive species face dramatic reductions in genetic diversity and the efficacy of selection; however, they are nevertheless able to spread and outcompete native species, a long-studied conundrum dubbed the paradox of biological invasions (Sax and Brown 2000; Frankham 2005; Schrieber and Lachmuth 2017). For invasive species with human preferences, the shortcomings associated with the classic paradox of biological invasions may not hold true if they were adapted to human environments prior to their invasion, as the need to adapt to a novel environment may be greatly diminished or completely eliminated (Lee and Gelembiuk 2008; Hufbauer et al. 2012). The idea that some invasive species had previously adapted to humans and their environments is especially crucial for understanding the history and genomic consequences of invasive range expansions in human diseases and their vectors (Hufbauer et al. 2012; Powell 2019; Comeault et al. 2020).

Over the course of an invasion, populations of invasive species face dramatic demographic changes through their introduction and along their expansion front (Sakai et al. 2001). These include introduction bottlenecks that reduce genetic diversity to an extent that depends on the severity of the bottlenecks and their source populations, the number of bottlenecks, and the amount of gene flow across the species range (Nei et al. 1975; Dlugosch and Parker 2008; Blackburn et al. 2016). The expected reduction in effective population size and subsequent loss of genetic diversity following the introduction of an invasive species will impact the species’ ability to adapt (Barton and Partridge 2000; Brandvain and Wright 2016). During a bottleneck, the lower effective population size reduces the efficacy of selection relative to drift, allowing weakly deleterious mutations to drift to higher frequencies, and will also decrease the probability that beneficial mutations will fix in a population. Species facing anthropogenic selective pressures might experience such strong selection that adaptation can proceed in spite of reductions in genetic diversity; however, adaptation will still be slowed if the introduced population's loss of diversity is severe enough that it will have to wait for new beneficial mutations (Orr and Unckless 2014; Osmond et al. 2019).

Importantly, the amount of gene flow between populations will also affect the efficacy of selection in contrasting ways: introducing new migrants to a population will increase the effective population size and thus the efficacy of selection, while the rate of influx of new mutations to a given locale can lead to a swamping effect, wherein selected alleles are overwhelmed by migration (Haldane 1956; Lenormand 2002; Yeaman 2022). For populations that are locally adapted, this gene flow is likely to primarily introduce maladapted alleles, but for populations with shared selective pressures like those from anthropogenic sources, gene flow may accelerate adaptation by introducing beneficial alleles that have arisen elsewhere in the global population that may not be present locally (Edelman and Mallet 2021; Yeaman 2022). In short, while theory predicts a decrease in the efficacy of selection and the rate of adaptation following a bottleneck and expansion, the degree to which this occurs in natural populations is less straightforward.

*Aedes aegypti*, the yellow fever mosquito, is a globally invasive species and the primary vector for the arboviruses dengue, yellow fever, chikungunya, and Zika. The species originated in Africa, where it spread during the African humid period, and differentiated into a generalist, *A. aegypti formosus* (hereafter *formosus*), and a human-adapted form, *A. aegypti aegypti* (hereafter *aegypti*), roughly 5,000 years ago (Brown et al. 2014; Rose et al. 2020; Rose et al. 2023). The *aegypti* form shows a higher preference for human hosts and an increased tolerance for rainfall variation, likely as a response to its strong association with humans (Rose et al. 2020). The *aegypti* forms are relatively poor dispersers by their own flight, but their association with humans leads to long-distance dispersal. Further, high egg desiccation tolerance and dormancy mean populations do not need to be immediately established but may emerge years after their initial introduction (Machado-Allison and Craig 1972; Fischer et al. 2019; Mayilsamy 2019), possibly with diverse lineages from these egg banks (Kaj et al. 2001; Evans and Dennehy 2005). *aegypti* has recently spread worldwide, first into the Americas shortly after European colonization and later into Asia (Brown et al. 2014). Worldwide *aegypti* populations appear to harbor far less variation than populations of *formosus* or those of mixed ancestry in Africa and potentially result from a single origin of human preference in Africa (Gloria-Soria et al. 2016; Lozada-Chávez et al. 2025). The expansion of *aegypti* out of Africa was likely complex, with high ship volume to the Americas during the human slave trade offering ample opportunities for multiple early introductions and continued gene flow (Powell et al. 2018). In recent decades, increasingly globalized trade may have allowed for additional long-distance dispersal events.

In the Americas, *aegypti* has had a substantial impact on human health and history (Tapia-Conyer et al. 2009; San Martín et al. 2010; Shepard et al. 2011; Cafferata et al. 2013). Outbreaks of yellow fever in the Americas were first reported in the 17th century (Blake 1968; Bryan et al. 2004), although they may have begun even earlier (Choppin 1878; Carter 1931), and continued outbreaks in the following centuries wreaked havoc on indigenous populations and naïve Europeans. Various epidemics of dengue, beginning in the 17th century (Brathwaite Dick et al. 2012), chikungunya, beginning in the 19th century (Brathwaite Dick et al. 2012), and more recently Zika in the 21st century (Chang et al. 2016) have occurred and continue to occur throughout the Americas, including the United States. During the 20th century, several countries in South America began an eradication program for *aegypti*, imposing intense pesticide pressure on populations, and by 1962, 18 South American and Caribbean countries had reported eradication (American Health Organization 1997). Recolonization, recrudescence from unidentified populations, or a combination of both led to the reemergence of the vector across the continent (Kotsakiozi et al. 2017). Insecticide resistance evolved rapidly and repeatedly throughout the Americas, with several haplotypes underlying resistance to multiple insecticides reported in the literature (Kawada et al. 2014; Al Nazawi et al. 2017; Haddi et al. 2017; Saavedra-Rodriguez et al. 2018; Fan et al. 2020; Love et al. 2023).

Introductions into the United States have been more recent, perhaps earliest in the Southeast. The first confirmed collection of the species was done in Savannah (GA) in 1828 (specimen cataloged as *Culex taeniatus* Wiedemann; Christophers 1960; Eisen and Moore 2013), but earlier outbreaks of diseases consistent with yellow fever in Spanish Florida in 1649 and the Northeast in 1668 suggest an earlier presence of the vector (Patterson 1992; Eisen and Moore 2013). These colonizations were then followed by subsequent spreads to the west, and multiple independent introductions reported in California with breeding populations were first reported in the state in 2013 (Pless et al. 2017). Despite close monitoring of recent introductions and efforts to monitor and control populations throughout the Americas, previous genetic studies of the species have primarily focused on individual regions, high-level descriptions of population structure, and/or have used limited genomic information. Currently, there lacks a detailed understanding of the demography of *aegypti* in North and South America, in particular the number and severity of *aegypti* introductions, the degree to which gene flow may aid in the spread and adaptation of populations throughout the continents, and how the efficacy of selection and the genetic load of introduced populations of *aegypti* have been affected by their demographic history.

Here, we examine a set of 131 whole genomes from African and American *A. aegypti* populations, encompassing the ancestral *formosus* and generalist *aegypti* forms, to model the demographic history of the species’ spread into the Americas, and investigate its effects on genome-wide diversity, the efficacy of selection, and genetic load. First, we investigate patterns of population structure across our dataset, within and among Africa and the Americas. We then infer the history of effective population size changes and split times between populations within and between the Americas and Africa. Finally, we investigate changes to the distribution of diversity in the introduced range, including genome-wide diversity, the distribution of fitness effects (DFEs) of new deleterious mutations, and the distribution of predicted deleterious alleles. We find that the spread of *aegypti* to the Americas appears to be best characterized by multiple apparent introductions and limited subsequent gene flow at fine and coarse geographic scales and that the history of bottlenecks and strong selection in the introduced range have had genome-wide impacts on diversity and the efficacy of selection. Despite a 33% to 40% reduction in neutral genetic diversity, *aegypti* in the Americas maintains high diversity and appears to have experienced only a modest reduction in the efficacy of selection. However, the complex demographic history has led to a simultaneous increase in the amount of deleterious alleles and some purging of strongly deleterious alleles in the *aegypti* form, especially in the Americas. The limited effect of its introduction history and recent anthropogenic selection on diversity and the efficacy of selection illustrate a surprisingly resilient species at the genomic level—one that poses a threat to future eradication efforts. We discuss the implications of this history and its genomic impact in the context of invasive species and vector control and adaptation (past, ongoing, and future) in the species.

## Results and Discussion

We used publicly available sequencing data from several studies, recently collated in the study by Love et al. (2023). This dataset includes 27 specimens from California and Florida (Lee et al. 2019), 18 from Santarém, Brazil, 13 from Franceville, Gabon, 19 from Kaya Bomu, Kenya, and 20 from Ngoye, Senegal (Rose et al. 2020), and 10 from Cali and 24 from Río Claro, Colombia (Love et al. 2023), for a total of 131 specimens, 79 of which are from the Americas. Hereafter, we refer to specimens sampled from the same location as accessions and to groups inferred to show evidence of recent shared ancestry as populations or clusters, depending on context. The African accessions used here were previously scored for human preference, with the Gabon accession exhibiting little-to-no human preference, the Kenya accession exhibiting intermediate/mixed preference, and the Senegal accession exhibiting strong human preferences (Rose et al. 2020), while the American accessions are assumed to exhibit strong human preferences.

### *Aedes aegypti* Is Highly Structured at Fine and Coarse Scales

**Fig. 1.**
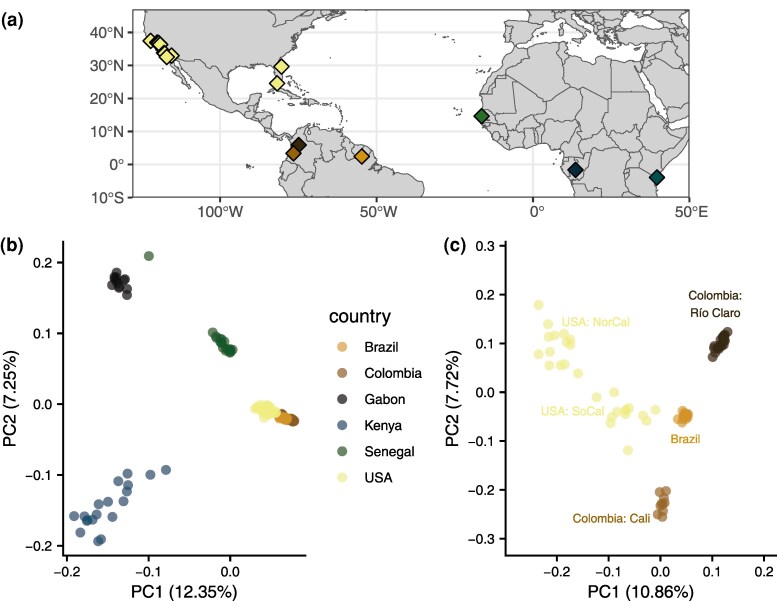
a) Sampling locations of specimens used in this study. b) PCA of all specimens highlighted by country of origin. c) PCA of American samples highlighted by location.

To characterize the population structure of our samples, we first used principal component analysis (PCA), beginning with our full dataset. The first and second principal components, representing 12.35% and 7.25% of the variance, respectively, separate the African accessions from the Americas and from each other (Fig. 1b; scree plot in supplementary fig. S1, Supplementary Material online). The African accessions separate distinctly from each other, with more apparent diversity in Kenya than in either Gabon or Senegal. The American samples cluster together, with the three South American accessions and the US accessions clearly differentiated within this regional cluster. Among the African samples, Senegal appears to be most closely related to the American samples and particularly to samples from the United States, consistent with previous studies identifying Senegal as a proxy for the ancestral *aegypti* form and with a more recent invasion in the United States. However, one Senegal specimen appears to cluster near to the Gabon cluster, which has been previously noted (Rose et al. 2020). This specimen could represent a recent migrant from a population closely related to the Gabon accession or a generalist individual within a larger-than-recognized range of *formosus*. PC1 is consistent with separating accessions by either latitude or host preference, and interestingly, PC2 primarily separates Kenya from Gabon and Senegal. Kenya and Gabon both exhibit primarily low human preference, with the former composed of mixed preference, evident here with the spread of the Kenyan sample along both PCs, with some specimens closer in PC space to American samples.

Within the Americas, principal components reveal more complex structure (Fig. 1c; scree plot in supplementary fig. S2, Supplementary Material online). Accessions from Colombia and Brazil separate clearly, with Brazil closer to either Colombian accession than the Colombian accessions are to each other, as previously reported (Love et al. 2023), despite being relatively close in geographical space (Fig. 1a); indeed, despite being separated by only ∼500 km, these two accessions are found nearly on opposite ends of PC2. As may be expected from geographically spread samples with few from a single location, the samples from the United States are loosely clustered into two groups in the first two PCs, and three to four groups apparent in deeper PCs (supplementary fig. S3, Supplementary Material online), similar to what was previously reported with these samples (Lee et al. 2019). The first US cluster in PC 1 and 2 groups Northern California and some accessions from Central California in the upper left of Fig. 1c. The second cluster, closer to Brazil in the center of Fig. 1c, consists of Southern California, a subset of accessions from Central California and Florida. In PCs 3 to 5, Central and Northern California clearly separate into groups: a cluster of Menlo Park with Madera and Fresno, and a cluster of Clovis and Sanger (supplementary fig. S3, Supplementary Material online). This separation is remarkable in that Clovis and Sanger are roughly 250 km apart and are on either side of Fresno with which they do not cluster. The Southern California and Florida cluster is largely maintained at PCs 3 to 5, with only the Florida sample from Vero Beach separating beginning at PC 4. The accessions from Exeter, CA, located in the Central Valley south of Fresno, cluster closely with an accession from Key West, FL, and near to the Southern California accessions. Similar to the Northern California cluster, structuring within the primarily Southern California cluster is hallmarked by a combination of fine and coarse-scale geographic separation and can likely be clustered into two to three groups within California and two separate Florida accessions.

**Fig. 2.**
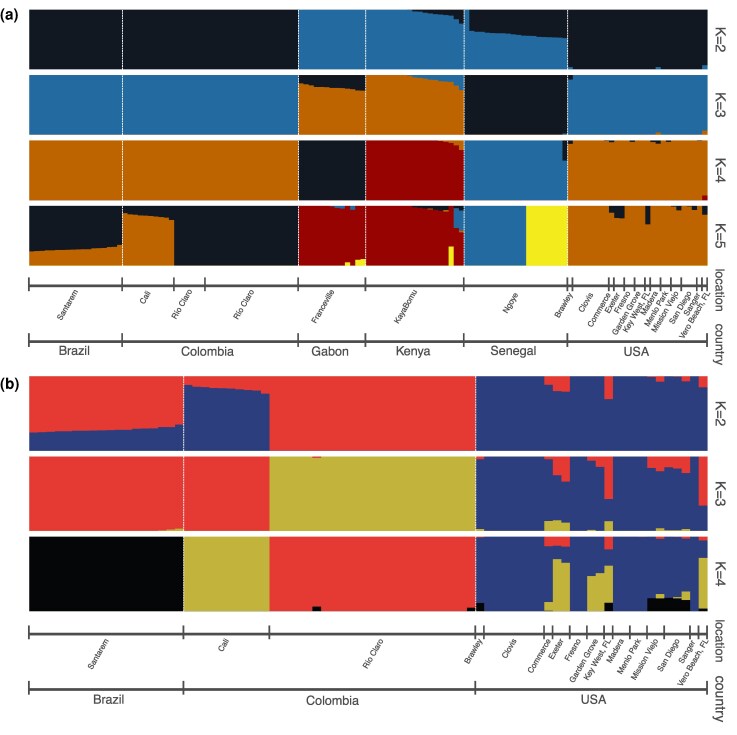
Admixture results for global samples a) and for the Americas b). The best *K*, obtained via cross-validation, is *K* = 2 for both a) and b), but we show results for higher *K* for additional context.

To more explicitly model population structure within our dataset, we used ADMIXTURE, which clusters samples using allele frequencies into a predefined number of groups, modeling each individual as a mixture of the clusters. Using the whole sample set, the best number of clusters according to cross-validation is *K* = 2, where samples are largely split into African and American groups (Fig. 2a). Several specimens in Kenya and nearly all in Senegal exhibit substantial admixture proportions, with the major component grouping with Gabon and the minor component with the Americas. All specimens from Gabon are entirely assigned to the same group (the major ancestry component in Africa), while the Brazilian and both Colombian accessions are all completely assigned to the opposing group—the minor ancestry component of Kenya and Senegal. Specimens from the United States are nearly entirely composed of ancestry shared with South America and the minor ancestry component of Kenya and Senegal. Three specimens, one of each from Southern California, Central California, and Vero Beach, FL, show small amounts of the Gabon-like ancestry.

Mixed ADMIXTURE group membership in Kenya and Senegal is consistent with domestic *formosus* ancestry or human preference previously reported in these accessions. Senegal, apart from one unadmixed specimen assigned entirely to the Gabon cluster, varies between a roughly 50:50 and 70:30 split of Gabon-like to South American–like group memberships, while about half of Kenyan samples have a small amount of South American–like ancestry. Higher values of *K*, although a slightly poorer fit according to cross-validation (supplementary table S1, Supplementary Material online), provide some context for the minor ancestry components in Senegal and Kenya. At *K* = 3 and above (Fig. 2a), the minor ancestry in Kenya groups with the American accessions, while Senegal largely appears unadmixed and groups with some ancestry in Gabon, or with itself, until *K* = 5 where it splits into two groups shared in minor proportions with some specimens in Gabon and Kenya. This is concordant with Senegal grouping most closely to the American accessions in PCA, but some Kenyan samples being nearly equidistant. While the extant accession in Senegal may represent the nearest known representative of an ancestral population to the American samples, these results from PCA and ADMIXTURE are consistent with ancestral population structure resulting ancestry shared among some African populations, including some domestic *aegypti* haplotypes that spread to the Americas (Slatkin and Pollack 2008). Back migration from the Americas to African populations (Brown et al. 2011; Powell and Tabachnick 2013) has also been proposed and would be consistent with low levels of American-like ancestry in Kenya during a second wave of the shift to human preference in African cities (Rose et al. 2020).

Within the Americas, the best number of groups according to cross-validation is again *K* = 2 (Fig. 2b), and the patterns of admixture are identical to the patterns in the Americas when considering all samples at *K* = 5 (Fig. 2a). At *K* = 2, accessions from Brazil and from Cali, Colombia, are shown as admixed, with reciprocal minor ancestry components, while Río Claro, Colombia, is unadmixed of the major ancestry component in Brazil. Greater grouping between Brazil and Río Claro is consistent with results from PCA (Fig. 1c) and divergence statistics (supplementary figs. S4 and S5, Supplementary Material online for genome-wide *F_ST_* and *D_xy_*) showing greater divergence within Colombia than between Río Claro and Brazil. The United States is represented as partially admixed and primarily composed of the major ancestry component in Cali. The admixture in the United States is isolated to the previously mentioned cluster present in the first two PCs of Southern California, FL, and the Central California accession of Exeter (Fig. 1c) and is present in some but not all of these individuals at *K* = 2, varying from minor to sizable proportions. At higher values of *K*, population structure remains qualitatively similar, with additional groupings adding to the diversity of admixture within the admixed US specimens, while the remaining US specimens compose their own group and South American accessions become unadmixed and quickly split among each other. This coarse-scale separation again reflects the two major clusters in the Americas, with little effect of geographic distance as evident in the separation within Colombia and the mixed fine and coarse-scale structure in the United States.

In summary, both ADMIXTURE and PCA results from the Americas depart strongly from expectations under a simple model of geographic isolation by distance. This may lend support to the possibility that there have been several independent introductions to the Americas, each sampling a subset of the ancestral *aegypti* variation available in Africa and little gene flow in the recent past. We examine these possibilities more directly in the section below.

### American *aegypti* Underwent Multiple, Strong Bottlenecks

**Fig. 3.**
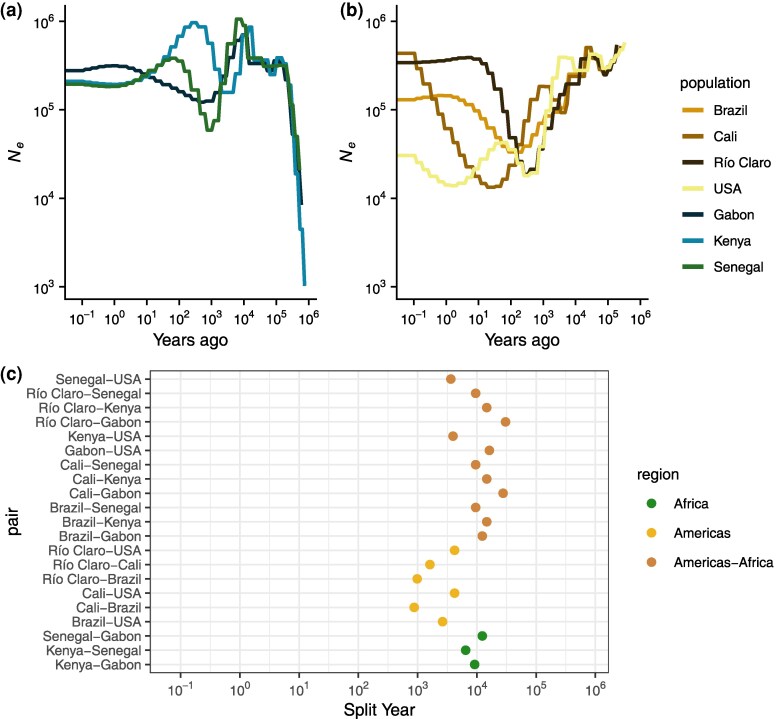
Estimated population size (*N_e_*) history and split times from SMC++. a) *N_e_* histories for all African accessions. b) *N_e_* histories for all American accessions. c) Split times for all accession pairs.

We sought to investigate the number and timing of introductions of *A. aegypti* among our sampled accessions, which represent locations of a range of possible events spanning from among the earliest to the most recent proposed introductions to the Americas. We began by inferring the historical effective population sizes among all countries using SMC++, making use of recent estimates for the generation time and mutation rate of *A. aegypti* (Rose et al. 2023). We report results obtained using the same model regularization parameter (rp = 5), which provided the most consistent fit across the seven groups examined in Fig. 3 as evidenced by coalescent simulations (supplementary figs. S8 to S14, Supplementary Material online). These analyses suggest that the ancestral effective population size (*N_e_*) among all accessions must have been between 300,000 and 400,000 until about 20,000 years (300,000 generations) ago (Fig. 3a and b). All accessions experienced a reduction in *N_e_* around 10,000 years (150,000 generations) ago, coinciding with the end of the African humid period as previously reported (Rose et al. 2023). Kenya and Gabon recovered to near ancestral *N_e_* over a span of a few thousand years, while Senegal appears to have suffered a deeper bottleneck than the other African accessions, dropping to roughly 80,000, with the lowest *N_e_* point around 1,000 years (15,000 generations) ago, before recovering to about 200,000. This bottleneck in Senegal occurred between 5,000 and 10,000 years ago, consistent with the founding and diversification of the *aegypti* domestic ancestry within Africa in the dry, variable Sahel after the African humid period (Rose et al. 2023). Stairway Plot 2, an alternative method, showed qualitatively similar patterns as those obtained via SMC++ in terms of the relative severity of bottlenecks across accessions (supplementary fig. S6, Supplementary Material online).

Among the American accessions, we infer strong, prolonged bottlenecks that vary in their timing and intensity. All American accessions began to decline between 5,000 and 10,000 years ago and continued to decline for thousands of years (Fig. 3b). In the accession from Cali, Colombia, we infer a fluctuating *N_e_* that was approximately stable until about 700 years ago, when its introduction bottleneck started and continued to decline until it reached a low *N_e_* of 13,312 only 20 years (300 generations) ago, followed by a recovery. The relatively larger *N_e_* of the Cali accession that is inferred at the time when other American accessions began to exhibit a contraction may suggest a more diverse sampling of lineages, possibly through multiple introductions or migration after colonization; both of these possibilities would result in a period of decelerated coalescence and are consistent with the higher degree of admixture inferred in Fig. 2b for this accession than the other South American accessions. In Río Claro and Brazil, bottlenecks beginning after the Africa humid period continued until their lowest *N_e_* points of 18,639 and 33,225 occurred 250 and 150 years (3,750 and 2,250 generations) ago, respectively. In all three accessions, we infer massive recoveries, to a present day *N_e_* of 433,679, 340,611, and 129,800 in Cali, Río Claro, and Brazil, respectively. Methods based on the sequentially Markovian coalescent (SMC) like SMC++ have limited ability to accurately estimate the *N_e_* in very recent time; however, the qualitative result of rapid recoveries in these accessions occurs within the span of generations in the past where these methods have reasonable accuracy (Patton et al. 2019), at least in Río Claro and Brazil, and is consistent with monitoring and disease prevalence suggesting a rebound after the collapse of Pan-American eradication efforts (reviewed in the study by Webb 2016), potentially through recolonization (Monteiro et al. 2014). While the bottlenecks in each locality are inferred to have occurred at different times over a span of roughly 200 years, we cannot be confident that they all represent different events; however, we can be confident that each of the South American accessions experienced strong bottlenecks and recoveries in the very recent past. Introduction bottlenecks in the South American accessions resulted in reductions in *N_e_* of 94%, 89%, and 96% in Río Claro, Brazil, and Cali, respectively, which appear to have occurred no later than 4,000 generations or 250 years ago.

### Population Structure Within the Americas

**Fig. 4.**
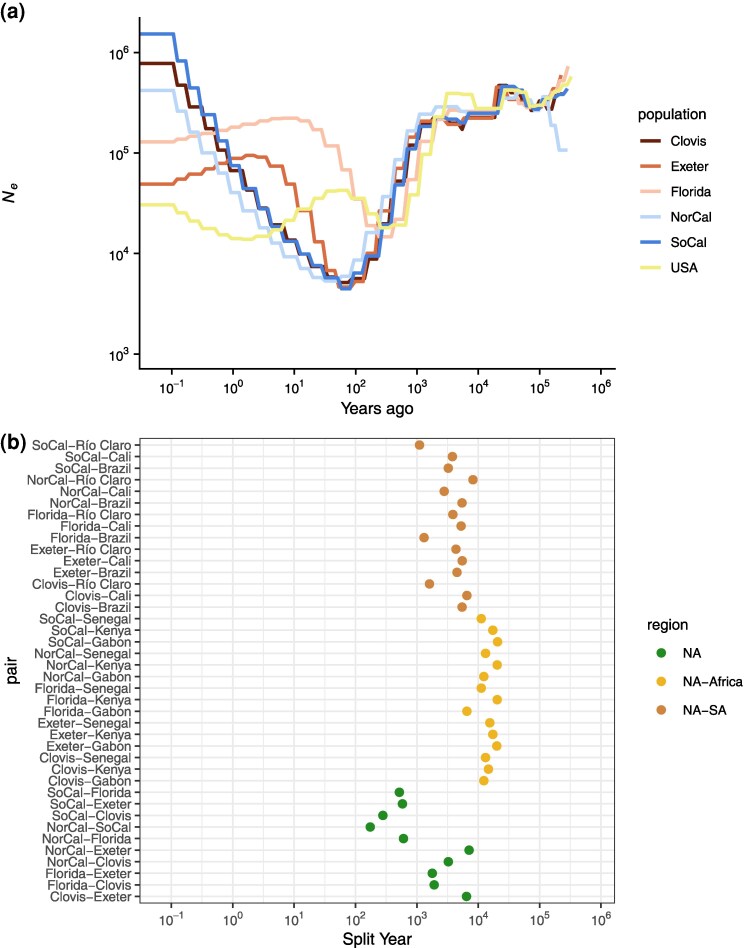
a) Effective population size histories for approximate US populations, with the estimated history of the combined accessions from the previous plot. b) Split times for all approximate US populations with all other accessions.

As evidenced by the PCA and ADMIXTURE results, the US accessions appear to represent a complex structuring of populations. However, grouping the US accessions into a single population when running SMC++ results in reductions in inferred *N_e_* similar to those in Cali, pointing to substantial bottlenecks in the underlying structured populations. We therefore split the United States into five approximate populations representing Southern California; Northern California; Florida; Clovis and Sanger, CA; and Exeter, CA; following clustering in PCA space (supplementary fig. S3, Supplementary Material online) and results from previous studies (Lee et al. 2019). All populations experienced a substantial bottleneck in the recent past, all beginning roughly 10,000 years ago as in the South American populations, but all peaking at a similar *N_e_* of about 5,000 only 55 years (825 generations) ago, except for Florida that peaked about 240 years ago at about 15,000 (Fig. 4a). All populations similarly are inferred to have recovered to between 400,000 and 1,500,000 at the present day, except for Florida and Exeter, which are inferred to have plateaued 5 to 10 years ago around 100,000, although this lower recovery may be an artifact of having only 2 samples in each of these populations. Similarly, the samples from Florida represent divergent lineages (supplementary fig. S3, Supplementary Material online), which may inflate *N_e_* estimates and bias the timing of bottlenecks. Among California clusters, the timing of the start and peak of each bottleneck is similar, though the Northern California cluster is slightly shifted to the more recent past, consistent with previous reports of a later, independent introduction relative to Southern California (Lee et al. 2019). As a whole, US populations experienced deeper and more recent bottlenecks than the South American populations, consistent with later introductions and an earlier introduction in Florida than in California, though the differences in *N_e_* between the US clusters are minimal. The apparent earlier introduction in Florida with a lowest *N_e_* roughly 240 years ago is after historical records of diseases matching the symptoms of Yellow Fever first appeared in Spanish Florida and the Northeast (Patterson 1992; Eisen and Moore 2013) but shortly before reports of the species in Georgia (Christophers 1960; Eisen and Moore 2013) and dengue outbreaks throughout port cities in the Eastern United States (Brathwaite Dick et al. 2012).

### Estimated Population Split Times Point to Deep Divergence

We also used SMC++ to infer population splits between all pairwise accessions. This model infers a split time using the within- and cross-population coalescent rates assuming a clean split model. At a broad scale, we find that split times involving African samples are older than split times within the Americas, though split times between Africa and the Americas span a range of time older and more recent than splits within Africa (Fig. 3c). Among the African accessions, Senegal and Kenya are inferred to be most closely related, followed by Kenya and Gabon, and Senegal and Gabon. This order of population splits is consistent with previously published behavioral results (Rose et al. 2020) and support the notion that the Senegalese and a portion of the Kenyan accessions appear to largely represent a derived domestic *aegypti* ancestry, while the Gabon accessions have maintained the ancestral generalist *formosus* form. The split times among the African accessions span a few thousand years, from 6,500 years ago for Kenya–Senegal, to 9,175 for Kenya–Gabon and 12,300 years ago for Senegal–Gabon. Assuming a single origin for human preference, mixed *aegypti* and *formosus* ancestry in the Kenyan accession would likely be the result of recent migration from an *aegypti* population more closely related to Senegal. It is important to note that in a clean split model not accounting for this recent migration, the estimated split time would be closer to the present, rather than correctly inferring an older split followed by recent migration. Alternatively, as previously mentioned, the domestic ancestry in these accessions may have sampled different diverse haplotypes in the ancestral domestic population, yielding the same signal, consistent with ADMIXTURE results. In either case, the split times presented here reflect a likely floor for the range of true split times.

Between Africa and the Americas, we infer a broad range of population split times. Among the South American accessions, we infer nearly identical split times in increasing order to Senegal (split times between the three South American accessions and Senegal were each estimated to be 9,545 years ago), Kenya (split times all estimated to be 14,678 years ago), and Gabon (27,699 years to Cali and 30,479 years to Río Claro), except for the BrazilGabon split, which is estimated to be slightly more recent than the Brazil–Kenya split (12,343 years ago; Fig. 3b). These split times far predate their proposed introduction to the Americas within the past 500 years and are on the order of or older than the splits within Africa, suggesting a deep split between extant African populations and the ancestral domestic population that eventually invaded the Americas. In contrast, split times between the United States and Africa are much more recent, though still far predating the American introduction (3,636 years ago with Senegal, 3,953 years ago with Kenya, and 16,256 years ago with Gabon); aside from a more recent split, this result could also be explained by migration between the African populations and the lineages leading to the US accessions after the split between the South American and African samples.

Within the Americas, estimated split times vary by thousands of years. In South America, our ordering of estimated population splits is consistent with results from PCA: Cali and Brazil most recently split (875 years ago), followed by Río Claro and Brazil (983 years ago), and finally Río Claro and Cali (1,613 years ago). These splits are well before the proposed introductions to the Americas, which occurred no earlier than roughly 400 years ago (Patterson 1992; Eisen and Moore 2013). Differences among these split times will reflect ancestral population structure but are likely inconsistent with a simple bifurcating tree, suggesting possible migration after their introduction. Regardless of any postintroduction migration, these estimated splits strongly suggest independent introductions, as the exclusion of migration in this framework is predicted to underestimate split times. Split times between South America and the United States are deeper still: estimated splits between the United States and both Río Claro and Cali are identical (4,203 years ago), while the split between the United States and Brazil is more recent (2,636 years ago). Again, the differences between these split times may reflect ancestral sorting and the extent of recent migration (if any). However, in this case, our estimates may also be affected by the presence of population structure in the United States.

With the potential impact of population structure in mind, we also considered split times for five different clusters of the US accessions to all other accessions (Fig. 4b). When considering these US partitions separately, the estimated split times are much deeper than when considering the United States as a whole. The splits between US clusters and African accessions are slightly older than those estimated between South American and African accessions. However, the split between Florida and Gabon is estimated to be substantially more recent at 6,536 years ago, potentially related to the more recent splits between Brazil and Gabon (Fig. 3b) and Florida and Brazil (Fig. 4b). For most US clusters compared with Cali and Brazil, the estimated split times are 4,500 to 6,500 years ago; however, Florida, Southern California, and Northern California have more recent split estimates, driving the US splits to these accessions as a whole toward the present. Southern and Northern California both have estimated splits to Cali about half as long ago as the rest of the United States (3,791 and 2,790 years, respectively), and Southern California also has a more recent estimate to Brazil (3,250 years ago). Florida stands out with a much more recent split estimate to Brazil (1,310 years ago), more recent even than its split times to Exeter and Clovis. While these estimates are much older than reasonable introduction times to the Americas, they may reflect a similar ancestral source population or an unsampled South American ancestral or admixture source population for Florida related to the accession presented here, especially in concordance with the high levels of admixture observed in the Floridian samples (Fig. 2b). In total, the estimated split times between the US partitions and those in South America do not suggest an origin for the sampled US accessions in related South American populations included in this study.

Within the United States, estimated split times again suggest a complex introduction history (Fig. 4b). There is a strong stratification in split times between a few US clusters—Florida and Northern California both have deep split times to the Central California accessions in Clovis/Sanger and Exeter, similar to the deep split between Clovis/Sanger and Exeter. These deeper splits span a broad range of time (1,785 to 7,100 years ago) with Florida more closely related to Clovis/Sanger and Exeter, while Northern California and Exeter is the deepest split. While deep splits between Florida and Central California may not be surprising and are consistent with independent introductions to California and Florida, the deep splits between some of the more geographically close California samples are more unexpected. For example, the accessions of Clovis/Sanger and that of Exeter are separated by about 80 km (split time estimate: 6,444 years ago) and Clovis/Sanger and Fresno (part of the Northern California cluster) are only separated by 16 km (split time estimate: 3,255 years ago). These findings suggest that there has been long-distance dispersal of related haplotypes within California, along with multiple introductions of lineages to several cities in the state that appear to have experienced little-to-no gene flow since their introduction and up until the time of sampling.

Among the more closely related set of accessions in the United States, estimated split times leave their origins less clear. The most recent split is between Northern and Southern California at 174 years ago, suggesting either an introduction from elsewhere within the Americas for both sets of accessions, as has been suggested at least for the Southern California populations (Lee et al. 2019), or a split somewhere within the Americas and subsequent dispersal to different regions. Southern California and the Clovis accession are estimated to have split similarly recently at 280 years ago, again suggesting they may have originated through a split from a population already within the Americas, or possibly long-distance migration along the California Highway 99 corridor. The remaining splits, between both Northern and Southern California and Florida, and Southern California and Exeter, occur within a timespan ranging from 519 to 606 years ago. While these timeframes are older than the presumed introduction, they remain much more recent than splits within South America or between American and African accessions, suggesting divergence within the Americas is not unlikely, but are also consistent with separate introductions followed by gene flow from the same source population.

### Demographic Models Suggest Migration and Deep Divergence

While results from SMC++ clearly suggest deep splits between sampled accessions in the Americas, this model does not allow for the possibility of migration between populations. To model demographic histories of these populations while explicitly considering the possibility for gene flow, we performed extensive demographic modeling in dadi (Gutenkunst et al. 2009). We inferred demographic parameters for an array of models with and without migration between both extant and ancestral lineages over combinations of three accessions, with each combination consisting of Senegal and two accessions from the Americas.

Comparing the top model fits across trios of accessions yields branching orders of populations consistent with those inferred using SMC++ (supplementary table S2, Supplementary Material online). However, the inferred times of these splits in generations are substantially deeper in time than those inferred with SMC++, as expected if there was gene flow that was accounted for in a clean split model. In particular, the top models suggest continuous migration on the order of 10^−6^ migrants per generation between most accessions, with migration between American accessions generally nearly an order of magnitude greater than either migration between an American accession and Senegal or between ancestral populations. In models with recent migration, split times within South America tended to be on the order of tens of thousands of years ago, while models without migration brought these estimates down to the range of a few thousand years ago, similar to SMC++ estimates in the absence of gene flow.

Overall, demographic modeling with dadi shows ordering of splits among accessions consistent with results from SMC++, though because most models include migration, split times tend to be much deeper than those inferred without gene flow in SMC++, as expected. Additionally, all models infer an ancestral *N_e_* of 400,000 to 800,000, consistent with estimates from SMC++. Together, through multiple lines of evidence, these demographic models agree upon deep divergence times among American accessions of *A. aegypti* that predate their introduction to the Americas and suggest a high likelihood of some migration among the introduced populations and between extant African lineages, consistent with previous results (Rose et al. 2020), though not likely in the recent past. Future work with deeper sampling of individual populations is needed to parameterize and time migration with high confidence.

### Introduced Populations Suffer Minimal Reductions in Selection Efficacy Despite Substantially Reduced Diversity

**Fig. 5.**
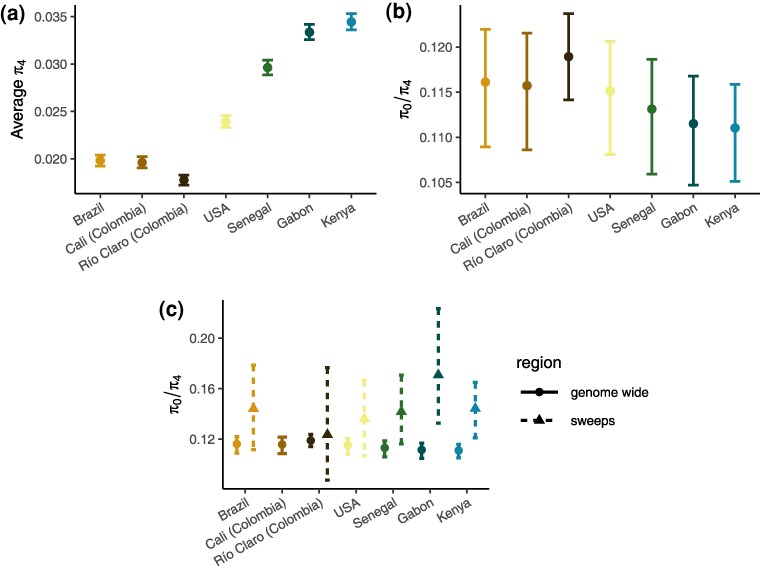
Diversity and measures of the efficacy of selection. a) Neutral diversity, *π*_4_. b) *π*_0_/*π*_4_ with bootstrapped errors for all accessions. American accessions show a subtle decrease in the efficacy of selection. c) *π*_0_/*π*_4_ in regions around sweep regions identified by Love et al. (2023) (note that Colombia was combined for sweep scans so is reported as a combined value for Río Claro and Cali; thus, *π*_0_/*π*_4_ estimates for these accessions include regions that may not necessarily be sweeps in both, and the inclusion of nonsweep loci could result in underestimates of *π*_0_/*π*_4_ for these accessions).

Given the strong bottlenecks experienced by all sampled introduced accessions, we sought to quantify the impact of the introduction history and previously described history of recent strong selection (Love et al. 2023) on genome-wide levels of diversity. We first estimated genome-wide neutral diversity using 4-fold degenerate sites (*π*_4_) (Fig. 5a). Among African accessions, neutral diversity ranged from about 0.03 in Senegal (95% confidence interval [CI]: 0.0289 to 0.0304) to nearly 0.035 in Kenya (95% CI: 0.0336 to 0.0353). This level of diversity is similar to that reported in *Anopheles gambiae* (Corbett-Detig et al. 2015) and somewhat higher than previous genomic estimates of all sites (Rose et al. 2020; Love et al. 2023). As expected given the demographic history of the accessions, synonymous diversity in the introduced range was much lower than in the ancestral range: nearly 0.02 in Brazil and Cali, Colombia (0.0198 and 0.0196; 95% CI: [0.0192 to 0.0204] and [0.0191 to 0.0202], respectively), and ∼0.018 in Río Claro, Colombia (95% CI: 0.0172 to 0.0183). Diversity in the combined US set of accessions was still <0.025 (95% CI: 0.0233 to 0.0246), despite being inflated by population structure. While a 33% to 40% reduction in diversity relative to Senegal is substantial, it is perhaps less of a reduction than expected given the large bottlenecks and eradication efforts sustained by these accessions. In an absolute sense and relative to other species (Leffler et al. 2012; Buffalo 2021), neutral diversity near 0.02 in the Americas is high, posing potential problems for future control efforts.

The combined effect of the introduction bottleneck(s) and recent selective pressures in the American *A. aegypti* populations should feed back into their ability to adapt because both of these phenomena are expected to reduce the efficacy of selection. We sought to test this using two indirect measures of the efficacy of selection. First, we estimated the ratio of 0-fold to 4-fold diversity in each accession (Fig. 5b). Assuming negative selection against new deleterious mutations is persistent, an increase in the *π*_0_/*π*_4_ ratio reflects a reduction in the efficacy of selection, i.e. an increase in the proportion of effectively neutral mutations as seen by selection has allowed weakly deleterious mutations to accumulate. We find African accessions to have a relatively low *π*_0_/*π*_4_ ratio (∼0.111 in Kenya and Gabon [95% CI: {0.1051 to 0.1159} and {0.1047 to 0.1168}] and ∼0.113 in Senegal [95% CI: {0.1059 to 0.1186}]). Consistent with a reduction in the efficacy of selection, the introduced accessions have higher ratios, 0.116 in Brazil and Cali (95% CI: [0.1089 to 0.1220] and [0.1086 to 0.1215], respectively) and 0.118 in Río Claro (95% CI: [0.1141 to 0.1237], while the grouped US accessions were near 0.115 (here artificially deflated due to population structure; 95% CI: [0.1081 to 0.1206]). These values are consistent with those reported in other insect species with no known history of introductions or eradication efforts (Chen et al. 2017). This decrease in the efficacy of selection is subtle, though perhaps unexpected given that neutral diversity in the introduced range remains high in an absolute sense, and in a relative sense was not as reduced as much as may have been expected.

**Fig. 6.**
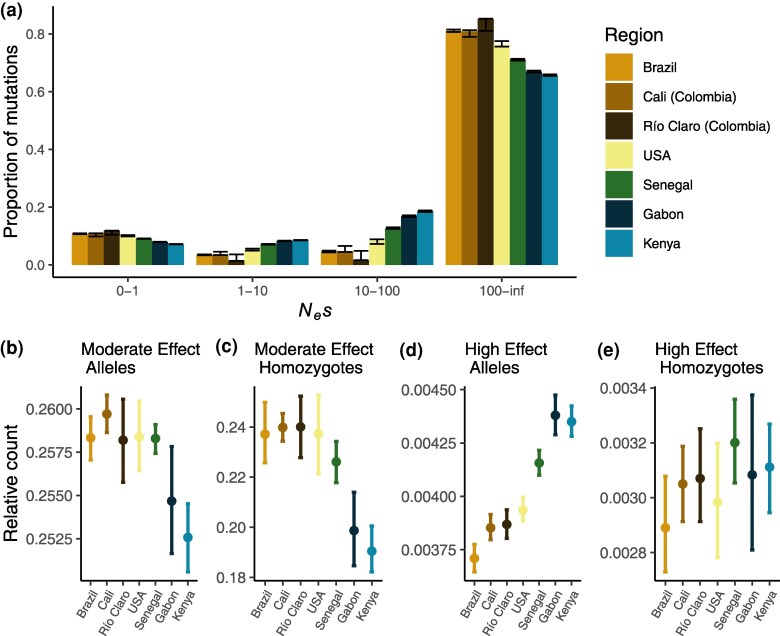
a) DFEs of new mutations for 0-fold degenerate sites relative to 4-fold degenerate sites for all regions, with error bars indicating 95% bootstrapped CIs. b) The ratio of the average number of moderate-effect deleterious alleles (as predicted by SnpEff) to the average number of derived synonymous alleles per individual. Error bars represent 95% bootstrapped CIs. c) Same as b) but showing the ratio of homozygous deleterious genotypes to synonymous homozygotes. d) Same as b) but for deleterious alleles predicted by SnpEff to have a “high” deleterious effect. e) Same as d) but for homozygous genotypes with a high predicted effect.

Inferring changes to the efficacy of selection via *π*_0_/*π*_4_ in nonequilibrium populations can be complicated by differences in the recovery time to equilibrium between nonsynonymous and synonymous alleles (Brandvain and Wright 2016). As such, an increase in *π*_0_/*π*_4_ of effectively neutral mutations as we find here may not solely reflect a reduced efficacy of selection. To address this, we can also use explicit modeling of the DFEs of new mutations. We therefore inferred the DFE for each accession, finding a clear difference between the African and American accessions: American accessions, and to a lesser extent Senegal, exhibit both an increase in strongly selected mutations and nearly neutral mutations (Fig. 6). The simultaneous shift to low and high *N_e_s* categories is evident in all introduced accessions, including the grouped US accessions where the effect is attenuated by population structure (Andersson et al. 2023). The shift is primarily driven by a reduction in the low and intermediate effect classes, with minor increases in the nearly neutral class and large increases in the strongly selected class. In the nearly neutral category, we see increases in the American accessions of 1.7% to 3% over Senegal and 3.5% to 4.5% over Kenya, representing a small but significant decrease in the efficacy of natural selection in introduced populations. Consequently, we do observe a small decrease in the efficacy of selection consistent with the increase in the ratio, though the biggest difference in the introduced accessions is in the unexpected shift toward more strongly deleterious mutations. However, it is possible that we have limited power to differentiate between moderately (10 to 100 *N_e_*s) and strongly deleterious mutations (100+ *N_e_*s) without knowledge of the true underlying DFE (Kousathanas and Keightley 2013); grouping these categories yields increases in the strongly selected categories for the American accessions of only 1% to 2% over the African accessions (supplementary fig. S7, Supplementary Material online).

### Introduced Populations Experienced Both Load Accumulation and Purging of Strongly Deleterious Alleles

While the *π*_0_/*π*_4_ ratio and the DFE can provide a general picture of the genome-wide changes to the efficacy of selection, they are indirect indicators that reflect long-term population trends. To more directly estimate the impact of potentially deleterious alleles and their differences between populations, we used annotations of the predicted effects of alleles from SnpEff (Cingolani et al. 2012) to estimate statistics of mean individual load. Specifically, we estimated both the mean number of alleles and the mean number of homozygous alleles of predicted moderate- and high-effect alleles per individual. These measures reflect how common potentially deleterious alleles are in each population and how they are presented within individuals. Because the visibility of alleles to selection depends on their dominance, which is unknown, we examine both of these measures to gain a broader understanding of the potential load in each population.

In Fig. 6, we show the ratio of the mean number of deleterious mutations per genome, as predicted by SnpEff, to the mean number of synonymous mutations per genome (the raw numbers of predicted deleterious mutations per genome are shown in supplementary figs. S15 to S17, Supplementary Material online). We find the ratio of moderate alleles to synonymous alleles in the Americas and in Senegal is increased relative to that in Gabon and Kenya, though is only significantly greater than Kenya (Fig. 6b). The ratio of highly deleterious to synonymous alleles, however, is significantly decreased in Senegal and all American accessions, with Senegal intermediate between the Americas and the other African accessions (Fig. 6d). We note that the absolute numbers of alleles of both classes follow similar trends (supplementary figs. S15 and S16, Supplementary Material online). Together, these results suggest that selection may have purged some highly deleterious alleles in populations of the human-adapted *aegypti* form, both in Senegal and in the Americas, while the demographic history of the American accessions likely increased the amount of moderately deleterious alleles per individual.

Although knowledge of the distribution of dominance in natural populations is limited, it is generally assumed that deleterious mutations are more likely to be recessive (Agrawal and Whitlock 2011; Huber et al. 2018), meaning that deleterious alleles are only visible to selection when they are homozygous. Considering this, we also measured the mean number of derived homozygotes per individual for alleles of different predicted selective effects. The ratio of highly deleterious homozygotes relative to synonymous homozygotes is not significantly different among populations, but the ratio of moderately deleterious homozygotes relative to synonymous homozygotes is significantly greater for all American accessions and Senegal (Fig. 6c and e). We note that the absolute number of predicted high-effect homozygotes is greater in Senegal and in the Americas, meaning that although there are fewer highly deleterious alleles in these populations, they are more likely to be exposed to selection. These results reflect the greater homozygosity in these populations (supplementary fig. S18, Supplementary Material online), suggesting that a larger number of weakly-to-moderately deleterious alleles may be exposed to selection in populations of the *aegypti* form and that selection has likely removed some highly deleterious alleles exposed as homozygotes.

### Regions Around Sweeps Experience Deleterious Draft

The efficacy of selection is additionally affected by selective sweeps (Smith and Haigh 1974), and in *A. aegypti*, particularly at sites under strong selection such as those underlying insecticide resistance (Love et al. 2023). While demography and selection both contribute to reductions in *N_e_*, selective sweeps only affect diversity in the regions linked to selected sites (Smith and Haigh 1974), meaning that the impact on both diversity and the efficacy of selection is heterogeneous along the genome. To elucidate the contribution of selective sweeps to the reduction in the efficacy of selection genome wide, we estimated *π*_0_/*π*_4_ in the 10 kb surrounding the top 1% of previously identified sweep signals (Love et al. 2023). We find the *π*_0_/*π*_4_ ratio is substantially increased in regions surrounding sweeps in all accessions (Fig. 5c). The Colombian accessions do not show as much of an increase as in other accessions, though this is likely due to the Colombian accessions having been grouped together in the previous work, dampening the effects of independent sweeps. This result is consistent with a substantial draft effect in the species shaping patterns of deleterious variation, as seen in other species (Chun and Fay 2011; Marsden et al. 2016; Wolff and Garud 2023), though here we are unable to quantify the amount of the genome affected. Future work is needed to determine the relative contribution of demographic and selective processes in shaping genome-wide diversity and the efficacy of selection, with important implications for *A. aegypti*'s evolved response to control efforts.

## Conclusion

Here, we investigated the demographic history of *A. aegypti* with respect to its introduction from Africa to the Americas. Our results suggest extensive population structure at fine and coarse scales, with some limited evidence for historical admixture in distant accessions in South America and between South America and some accessions in the United States, while many accessions in close proximity exhibit deep divergence with no evidence of recent admixture. In particular, accessions from Colombia and Brazil appear to be largely genetically distinct, with the accession in Cali, Colombia, more closely related to the accession from Santarém, Brazil, than to the accession in Río Claro, Colombia, despite the relatively short geographic distance between the Colombian sampling locations. Similarly, accessions in California overlap each other in geographic space in the Central Valley, despite having estimated divergence times hundreds to thousands of years ago. The geographic overlap near Fresno, California separates the broad Northern California genetic cluster from the Southern California cluster, the latter of which includes accessions from Florida, further highlighting the complexities of scale in population structure of the species. Together, our observed patterns of population structure and admixture imply that gene flow between regions may be limited, but occasional new (or re-) colonizations likely occur through long distance dispersal, as is likely within California. Our admixture results are more broadly consistent with the sorting of diverse ancestries during the introduction bottlenecks, supported by deep estimated divergence times. In the context of the spread of beneficial mutations, such as those conferring insecticide resistances, our results suggest that adaptation may occur through parallel mutation instead of through the spread of single-origin mutations across large geographic ranges, though future work is needed to more directly test this hypothesis at loci of interest.

Regarding the number and timing of introductions in the Americas, we find through inferences of historical effective population sizes and population split times that populations in both South and North America likely originated through multiple introductions. We note that inferring the precise timing of introduction events with genomic data is challenging, and our estimates should not be treated as exact; however, our divergence estimates suggest American populations split thousands of years ago, long before the proposed introduction of *aegypti* to the Americas via the slave trade (Rose et al. 2023). Similarly, while the differences in bottleneck strength and timing, the general lack of admixture in the Americas, and estimated split times predating possible introduction times by hundreds of years strongly suggest there have been multiple origins in South America, we cannot directly ascertain whether populations that were declared eradicated were in fact eliminated and recolonized from elsewhere or if instead recovery occurred from the expansion of a smaller number of individuals surviving in refugia. However, the fact that we do not observe especially strong bottlenecks that would be expected for a population recovering from near eradication, perhaps makes the recolonization hypothesis more likely. Similarly, for accessions in the United States, estimated split times and bottleneck severities and timings point to independent origins relative to the sampled South American accessions, although we cannot identify the introduction route with the geographic sampling in this study. Notably, some pairs of North and South American clusters exhibit markedly more recent estimated split times than other such pairs (e.g. Southern California and Clovis relative to Río Claro and Florida relative to Brazil). These more recent split time estimates, which we obtained under a model that does not allow for migration, could potentially be underestimates caused by postsplit gene flow between some North and South American clusters; this notion is consistent with our evidence of relatively low but nonzero admixture proportions shown in Fig. 2b and in results from dadi modeling. Likewise relative to African accessions, the markedly recent split time estimates between both Florida and Brazil from Gabon could reflect recent gene flow to the Americas, back migration to Africa, or shared admixture in Florida and Brazil from Gabon that predates their introduction to the Americas. Future work is needed to parse out the unique specifics of gene flow and American introductions of individual groups of samples.

We find all introduced accessions have similar levels of diversity, reduced by only ∼33% to 40% from the ancestral levels. Diversity in these accessions therefore remains high (*π* ≈ 0.02 at synonymous sites), reflecting a model where *N_e_* only dropped to the tens of thousands in South American populations before quickly recovering. We note that population structure in the United States means the diversity estimates and measures of the efficacy of selection we report here are inflated, with underlying populations possibly exhibiting lower diversity than in South America given the recent low *N_e_* in each US accession of about 5,000 (Fig. 4a). Nevertheless, diversity levels in the United States when grouped into a single population are still estimated to be reduced relative to ancestral levels. We additionally find evidence of only subtle reductions in the efficacy of selection relative to the ancestral range, as reflected in an increased *π*_0_/*π*_4_ and an increase in the proportion of weakly deleterious mutations in the DFE.

Despite evidence for only a subtle decrease in the efficacy of selection, we find significant impacts to the genetic load of introduced populations. Compared to the African accessions, accessions from the Americas show an increased abundance of alleles predicted to have moderately deleterious effects, but also a reduction in the abundance of predicted strongly deleterious alleles, relative to the number of synonymous polymorphisms. All derived alleles appear to be more commonly found in the homozygous state in the introduced populations, and although this may in part be due to demographic changes, the fact that American accessions show an excess ratio of predicted moderately deleterious mutations to homozygous synonymous alleles suggests that there has also been a reduction in the efficacy of selection. However, American accessions exhibit a lower ratio of homozygous strongly deleterious mutations to homozygous synonymous mutations than observed in Africa (with the human specialist samples from Senegal showing an intermediate value of this ratio), suggesting that selection has more efficiently purged strongly deleterious alleles from these populations. The respective excess and deficit of homozygous moderately and strongly deleterious alleles in the introduced populations are consistent with our estimated U-shaped DFE. This pattern has been previously predicted in simulations of population expansions (Gazave et al. 2013), suggesting the bottleneck-and-expansion history of *aegypti* populations may have simultaneously reduced the efficacy of selection on weak-to-moderately deleterious alleles, but the increased homozygosity experienced by these populations (supplementary fig. S18, Supplementary Material online) has allowed for more efficient purging of strongly deleterious alleles, which are likely to be more recessive than more weakly deleterious alleles (Huber et al. 2018). This distribution of deleterious variation in the introduced range may inhibit adaptation through Hill–Robertson interference (Hill and Robertson 1966; Castellano et al. 2016). Future work is needed to further characterize the dynamics of selected alleles and the consequences of accumulated load on these dynamics in the *Ae. aegypti* genome.

We do additionally find evidence that the efficacy of selection around selective sweeps is similar between African and American accessions, suggesting that the genome-wide signal of a weakly increased *π*_0_/*π*_4_ in the Americas may be due primarily to the demographic history, though future work is required to tease apart the relative contributions of demography and selection to the genomic landscape of diversity in the species, and how deleterious variation may be distributed. This genomic resilience given the introduction bottlenecks, rapid expansions, and history of eradication attempts underscores the challenge faced by ongoing and future control efforts. Despite their demography, American populations of *A. aegypti aegypti* all exhibit high diversity and a strong efficacy of selection, making the path of rapid adaptation to ongoing and future anthropogenic control efforts likely even in the absence of strong gene flow.

## Materials and Methods

### Data Filtering

Here, we make use of a previously curated and filtered whole-genome sequencing dataset of 131 samples from (Love et al. 2023), which includes 27 samples from California and Florida from (Lee et al. 2019), 18 samples from Santarém, Brazil, 13 samples from Franceville, Gabon, 19 samples from Kaya Bomu, Kenya, and 20 samples from Ngoye, Senegal, taken from the study by Rose et al. (2020), as well as 10 samples from Cali, Colombia, and 24 samples from Río Claro, Colombia. We used the VCF (Variant Call Format) from (Love et al. 2023), with detailed methods for alignment, variant calling, and filtering described therein. Briefly, Colombian samples were sequenced using paired-end 150-bp Illumina reads, quality checked with FASTQC 0.11.9 (Andrews 2010), and trimmed with Trimmomatic 0.39 (Bolger et al. 2014). Reads from all specimens were aligned to the AaegL5 reference genome using bwa-mem2 version 2.1 (Vasimuddin et al. 2019) and then genotyped with GATK HaplotypeCaller version 4.1.9.0 (DePristo et al. 2011; Poplin et al. 2018), using the EMIT_ALL_CONFIDENT_SITES flag. SNPs (Single Nucleotide Polymorphisms) were filtered using scikit-allel version 1.3.2 (DOI: 10.5281/zenodo.4759368). The dataset from the study by Love et al. (2023) that we used here imposed the following filters:

1. For the Colombian accession, specimens with mean coverage below 15× or fewer than 70% of reads mapping were removed.
2. For the full sample, indels were removed, and SNPs were filtered following GATK's best practices (https://gatk.broadinstitute.org/hc/en-us/articles/360035890471-Hard-filtering-germline-short-variants). Specifically, sites were retained if they passed five of the six following filters: (i) variant quality by depth ≥2, (ii) variant strand bias via Fisher's exact test <40, (iii) variant strand bias via symmetric odds ratio test <4, (iv) mapping quality (MQ) ≥40, (v) MQ rank sum test between −5 and 5, inclusive, and (vi) site position within reads rank sum test between −3 and 3, inclusive.
3. All SNPs that were not called in at least 75% of specimens in at least five of the six countries were removed.
4. All nondiallelic SNPs were removed.
5. We removed regions previously identified as repetitive (Matthews et al. 2018) and regions that were not uniquely mappable.

In addition to these filters used by Love et al. (2023), for analyses that required regions of the genome not likely to have experienced strong linked or direct selection (see sections “Population Structure and Admixture” and “Historical Effective Population Size Inference and Split Times), we further filtered the genome to remove all sites within 100 kb of annotated genes and for locations of centromeres as reported in the study by Matthews et al. (2018). This mask is referred to as the intergenic sites mask in later analyses.

For diversity statistics that require an accurate count of quality invariant sites, we further removed invariant sites with more than 25% of samples missing base calls using bcftools version 1.16 (Danecek et al. 2021), as well as sites with less than a depth of 4 or greater than a depth of 30. All subsetting or filtering of VCFs for specific masks or regions were performed using bedtools v2.30.0 (Quinlan and Hall 2010) unless otherwise noted. Some analyses required sites of 0- or 4-fold degeneracy, which were called using a python script (https://github.com/tvkent/Degeneracy), the L5.0 version of the *A. aegypti* genome and annotation, NCBI accession GCF_002204515.2 (Matthews et al. 2018).

### Population Structure and Admixture

To infer the population structure of *A. aegypti,* we used two different approaches. First, we used PCA clustering as a high-level description of the underlying structure. We used the bedtools tool *intersect* to remove sites in the aforementioned intergenic sites mask from the full VCF file and converted to plink format using the --make-bed function in plink v1.90b3.45 (Purcell et al. 2007). Sites were thinned for linkage disequilibrium (LD) by removing sites in 100 kb windows exceeding an *r*^2^ of 0.5, with a step size of 10 kb. Principal components were then calculated with plink for the full dataset and separately for samples from the Americas. Second, we estimated population structure more explicitly with ADMIXTURE (Alexander et al. 2009), using the same LD-pruned SNP set for all samples and for the Americas with *K* = 2 to 10. The *K* number of populations with the best predictive accuracy was determined using ADMIXTURE's cross-validation with the --cv flag, and we present results for several *K* around this value.

### Historical Effective Population Size Inference and Split Times

In order to obtain a view of the history of bottlenecks and expansions in *A. aegypti*, we inferred the historical effective population size of each accession. Inference of historical *N_e_* makes use of information in the site frequency spectrum (SFS) (Liu and Fu 2020) and/or uses local sequence information to infer a distribution of coalescent times with the SMC (Mather et al. 2020). SMC-based methods have been shown to have the highest accuracy in the deep past, while SFS-based methods are most accurate in the relatively recent past (Liu and Fu 2015; Patton et al. 2019; Liu and Fu 2020), only when there is a large enough sample size (Terhorst and Song 2015). Because of our sample size constraints, unphased data, and the lack of an unfolded SFS, we use SMC++ (Terhorst et al. 2017), which has been shown to have high accuracy for a broad time frame (Terhorst et al. 2017; Patton et al. 2019) including the recent and distant past, through use of both the SMC framework and incorporating information on the SFS from additional individuals in the sampled population. We also provide inferences from the SFS-based Stairway Plot 2 (Liu and Fu 2020) in the Supplementary material online for qualitative assurance of size histories. For Stairway Plot 2, we used intergenic sites 10 kb away from genes. For each method, we scaled generations to years and set the per generation mutation rate using estimates previously reported in (Rose et al. 2023): 0.067 years per generation and a mutation rate of 4.85 × 10^−9^.

We used SMC++ v1.15.4 (Terhorst et al. 2017) and masked the genome using our intergenic sites mask. We ran inference on all accession as described in the previous section, as well as dividing the United States into five putative populations determined by their clustering in PCA space and corroboration with (Lee et al. 2019): Southern California (Commerce, Mission Viejo, Garden Grove, Brawley, and San Diego), Northern California (Madera, Menlo Park, and Fresno), Clovis and Sanger, Exeter, and Florida (see Supplementary material online for plots of PC's 1 to 5). We converted a VCF for each chromosome to SMC++ format with the vcf2smc function in SMC++ using our mask and choosing random individuals as “distinguished” lineages. SMC++ makes use of the information captured in multiple samples by combining the coalescent times and allelic states between haplotypes of a set of one or more “distinguished” individuals, as in other SMC methods, with the allele frequencies of a set of “unlabeled samples” conditioned on the information from the distinguished individuals (Terhorst et al. 2017). For accessions with more than five samples, we used five distinguished individuals; for accessions with three to five samples, we used two distinguished individuals; and for accessions with only two samples, we chose a single distinguished individual, with the remaining individual specimens from an accession considered as unlabeled samples. We ran the estimate function in SMC++ on these data, using 10 knots, a window size of 10, and regularization penalties of 4, 5, and 6 for inference over the past 800,000 years. Because the choice of regularization penalty resulted in qualitatively different N_e_ trajectories for some accessions (see “Results”), we ran neutral simulations in *msprime* version 1.2.0 (Baumdicker et al. 2022) to estimate the site frequency spectra under the inferred demographic histories to independently compare the fit of SMC++ runs under the three different regularization parameters. Simulated SFSs were extracted from the simulated tree sequence using tskit version 0.5.6 (Kelleher et al. 2019) and compared to the observed SFS for each accession using a multinomial likelihood calculation following (Beichman et al. 2017); we present results for all accessions with a regularization penalty of 5, which was a better fit for our South American accessions and Gabon, and did not qualitatively alter the results for Kenya and Senegal (see “Results” and supplementary figs. S8 to S14, Supplementary Material online). We used the same parameters for the USA accessions, though we did not assess the fit of the SFS for these accessions as the sample sizes are too low to reliably use SFS-based methods and following the other accessions, the qualitative results are unlikely to be altered.

SMC++ also allows for estimation of population split times via a clean split model using inferred within- and cross-coalescent rates from SMC++ inference within and between populations. To estimate split times between accessions, we used the split function in SMC++ for every accession pair. We followed the same procedure described above to determine distinguished individuals for each accession. We then converted a VCF for each chromosome to SMC++ format using the vcf2smc function, and separately for each accession using the distinguished individuals in the focal accession. We treated all remaining individuals from both accessions as undistinguished lineages. We then ran the split function in SMC++ using these joint SMC++ files and the model fits for each accession from the previous estimate step.

Finally, we ran Stairway Plot 2 (Liu and Fu 2020) on the country-level groups of accessions (splitting Colombia into Río Claro and Cali) as an independent estimate of the *N_e_* trajectories. We used an intergenic site SNP set filtered as described in the section “Data Filtering,” but masking 10 kb around genes. For each accession, we calculated the folded SFS using scikit-allel (DOI: 10.5281/zenodo.4759368) and then ran Stairway Plot 2 using the mutation rate and generation time estimates from the study by Rose et al. (2023) as with SMC++, and a sequence length, *L*, of 66,563,773, representing the number of filtered variant and invariant sites used to generate the SFS.

### Demographic Modeling

We used a combination of donni (Tran et al. 2024) and dadi (Gutenkunst et al. 2009), to explicitly model demography with and without migration. Although dadi allows for up to five populations in a model, we chose to model three accessions at a time due to the computational constraints imposed by a greater number of populations, hereafter “trios,” using combinations of Senegal and two American accessions in order to build a composite picture of demography across all accessions.

To get starting parameters and to narrow the number of models we fit, we performed a first pass by considering all 3D populations available in the dadi_pipeline package v3.1.7 (Portik et al. 2017) and estimating their demographic parameters for all trios using the neural network package donni. We obtained folded 3D SFSs in dadi format using the intergenic sites VCF with easySFS (https://github.com/isaacovercast/easySFS) (Gutenkunst et al. 2009), with projections set at 80% of each population's haploid sample size. We used the infer command in donni to download and use pretrained models to estimate parameters for these models from the observed 3D SFSs. Several models for each trio did not find parameters that fit, suggesting poor model choice, and these models were not considered for further analysis. Models that did estimate parameters were visually evaluated for their residuals, and models with obviously large residuals were also removed. Estimated parameters for 100 models with reasonable residuals were used as starting parameters for refined model fitting in dadi.

Models run in dadi were given as starting parameters the estimated model parameters from donni, with lower and upper bounds three orders of magnitude below and above each parameter, respectively. Projections were again set to 80% of the haploid sample size of each population. Inference was run using a modified version of the dadi_Run_3D_Set.py script provided in dadi_pipeline v3.1.7, using the intergenic sites VCF as input to obtain the folded 3D SFS natively within dadi. Briefly, dadi_pipeline uses four rounds of optimization of dadi using the Nelder–Mead method with, respectively, 10, 20, 30, and 40 reps, maxiter set to 3, 5, 10, and 15, and 3, 2, 2, and 1 folds (Portik et al. 2017). Final model fits were checked for low and evenly distributed residuals, and models within trios were ranked by log-likelihood and compared for similar parameter estimates.

### Diversity Statistics and the DFEs

We calculated pairwise genetic diversity as *π* for each accession using *pixy* (Korunes and Samuk 2021) in windows of 500 kb, following the study by Love et al. (2023), separately for all sites, 0-fold sites, and 4-fold sites. Genome-wide averages were recalculated using the raw *pixy* output as the sum of differences (count_diffs) divided by the sum of comparisons (count_comparisons). We also report genome-wide average *F_ST_* and *D_xy_* for all sites (supplementary figs. S4 and S5, Supplementary Material online). Error bars for *F_ST_* and *D_xy_* were calculated as the 95% bootstrapped CI with 100 bootstraps over windows in R. We further report the average genome-wide homozygosity at SNPs, estimated with scikit-allel, with 95% bootstrapped CIs estimated with 100 replicates in python (supplementary fig. S18, Supplementary Material online). *Aedes aegypti* has faced substantial eradication efforts, and as such has undergone strong, recent positive selection (Love et al. 2023), which should lead to genomic heterogeneity in the efficacy of selection due to hitchhiking (Smith and Haigh 1974). As such, we also estimated the ratio *π*_0_/*π*_4_ in regions immediately surrounding selective sweeps. For diversity statistics around selective sweeps, we filtered the Sweepfinder results from the study by Love et al. (2023) for the top 1% of CLR windows and calculated the statistic of interest in windows surrounding these 10 kb outlier windows.

Because the behavior of statistics like *π*_0_/*π*_4_ is unclear in nonequilibrium populations, we additionally estimated the DFEs of new mutations in each accession, which is thought to be more robust to nonequilibrium dynamics (Brandvain and Wright 2016). To estimate the DFE, we first calculated the folded SFS using the sfs_folded function in scikit-allel for 0-fold and 4-fold sites. We estimated the DFE for 0-fold degenerate sites relative to 4-fold degenerate using DFE-alpha version 2.15 (Keightley and Eyre-Walker 2007) using default parameters and obtained *N_e_*s bins using the prop_muts_in_s_ranges function. Ninety-five percent CIs were calculated by bootstrapping the site frequency spectra by site using the random.choice function in numpy (Harris et al. 2020) to randomly sample the genotype arrays and reestimate the DFE with 1,000 replicates.

### Estimates of Genetic Load

As a more direct estimate of the genetic load in each population, we predicted the effects of new mutations in coding regions of the genome using Snpeff (Cingolani et al. 2012). We used the “ann” function of Snpeff version 5.2c to annotate mutational effects of our full VCF with the Aedes_aegypti_lvpagwg database provided by Snpeff. Because Snpeff annotates predicted deleterious function relative to the reference genome, which is an unrelated *A. aegypti* strain that has been maintained in lab conditions for decades, the deleterious allele at a given site must be polarized relative to an assumed ancestral population. Here, we used the intersection of the Gabon and Kenya populations, which are primarily of the presumed ancestral generalist form, to polarize the reference and alternate alleles as major and minor, with the intersection of minor alleles in both populations presumed to be derived and/or deleterious. We then estimated the deleterious load under two assumptions of dominance using sites annotated as having “HIGH,” “MODERATE,” or either of these two predicted severities, as well as sites annotated as synonymous as a null expectation considering the demographies. The relevant effects of deleterious alleles depend on their dominance, which is unknown. Thus, we estimated load at all focal sites assuming additivity by counting the number of annotated deleterious alleles per individual and at all focal sites assuming full recessivity by counting the number of homozygous genotypes of the annotated deleterious allele per individual in each population. We used scikit allel version 1.3.2 (DOI: 10.5281/zenodo.4759368) to polarize and count alleles, and estimated percentile bootstrap 95% CIs of the mean values per population using the bootstrap function of scipy version 1.10.0 (Virtanen et al. 2020), resampling over sites with 1,000 replicates. Because the count of synonymous alleles will also change after demographic events like population size changes, we additionally calculated the ratio of these metrics for a given class of predicted deleterious alleles relative to synonymous sites. As our point estimate we calculated the ratio of the mean deleterious count over the mean synonymous count, and estimated 95% CIs by taking the ratio of the full bootstrap distribution of each site type and estimating the 2.5% and 97.5%iles.

## Supplementary Material

evaf066_Supplementary_Data

## Data Availability

All sequencing data used in this paper are publicly available (see Love et al. 2023). All code used for the analyses presented in this paper can be found at https://github.com/tvkent/aedes_demography/.
